# To What Extent Can Digital Health Technologies Comply With the Principles of Responsible Innovation? Practice-and Policy-Oriented Research Insights Regarding an Organisational and Systemic Issue

**DOI:** 10.34172/ijhpm.8061

**Published:** 2024-10-21

**Authors:** Hassane Alami, Pascale Lehoux, Sara E. Shaw, Marietou Niang, Kathy Malas, Jean-Paul Fortin

**Affiliations:** ^1^Department of Health Management, Evaluation and Policy, University of Montreal, Montreal, QC, Canada.; ^2^Center for Public Health Research of the University of Montreal, Montreal, QC, Canada.; ^3^Institute for Data Valorization (IVADO), Montreal, QC, Canada.; ^4^Nuffield Department of Primary Care Health Sciences, University of Oxford, Oxford, UK.; ^5^Université of Quebec at Rimouski, Levy, QC, Canada.; ^6^Executive Office & Research Centre of The University of Montreal Hospital, Montreal, QC, Canada.; ^7^Department of Entrepreneurship and Innovation, Montreal Business School (HEC), Montreal, QC, Canada.; ^8^Faculty of Medicine, Laval University, Quebec, QC, Canada.; ^9^VITAM Research Centre on Sustainable Health, Faculty of Medicine, Laval University, Quebec, QC, Canada.

**Keywords:** Digital Health, Responsible Innovation, Health Technology Assessment, Health System, Sustainability, Equity

## Abstract

**Background:**

Digital health technologies (DHTs) have expanded exponentially since the COVID-19 crisis and have prompted questions about their impact across all levels of health systems. Because health organisations and systems play a central role in the success or failure of the transition to more equitable and sustainable societies, the concept of Responsible Innovation in Health (RIH), focused on aligning the processes and outcomes of innovation with societal values, is gaining interest in research, policy, and practice. This study aims to explore enablers and constraints to the development, procurement and/or utilisation of responsible DHTs in health organisations.

**Methods:**

Semi-structured interviews were conducted with 29 stakeholders concerned with the development, procurement, and/or utilisation of DHTs in a large Canadian academic health centre. Data were thematically analysed through a mixed deductive-inductive process using the RIH framework.

**Results:**

Our findings highlight that the consideration of RIH principles in the development, procurement, and/or utilisation of DHTs depends mainly on organisational and systemic factors and conditions, namely: (1) the presence of an organisational culture that promotes RIH in its innovation-related practices and processes; (2) availability of material and financial resources as well as expertise in certain fields (eg, environmental sustainability); (3) the evolution of health technology assessment (HTA) practices to include other dimensions beyond effectiveness, safety, and costs; (4) the scope of the regulatory and legal frameworks that govern the approval and use of DHTs; and (5) the role of the market (eg, venture capital) in the design of federal and provincial innovation policies.

**Conclusion:**

This study provides insights on practice, policy, and political issues that health organisations may face in the development, procurement, and/or utilisation of responsible DHTs. It can help scholars, practitioners, decision-makers, and industry to create the conditions for a better integration of RIH principles into health organisations and systems.

## Background

**Key Messages**

**Implications for policy makers**

- Digital health technologies (DHTs) are seen as a lever to improve healthcare. However, they can also contribute to exacerbating health inequalities, threaten the financial sustainability of health systems, and contribute to pollution and climate change.
- Responsible Innovation in Health (RIH) aims to align the processes and outcomes of innovation with societal values and guide researchers, practitioners, decision-makers, and industry in the implementation of responsible, equitable, and sustainable DHTs.
- Beyond a spontaneous interest towards RIH, the ability to include the principles of RIH in the development, procurement, and/or utilisation of DHTs depends on key practical, clinical, organisational, regulatory, economic/financial, and socio-political factors and conditions reflecting the complexity of health systems.

**Implications for the public**

Digital health technologies (DHTs) are generating considerable enthusiasm and expectation in their potential to transform 21st century health systems. At the same time, the way they are designed, developed, promoted, marketed, implemented, and used raises major ethical, human, socio-political, regulatory, economic/financial, and environmental issues. Responsible Innovation in Health (RIH) can help to ensure that DHTs benefit the entire population, without discrimination, while contributing to more sustainable health systems that are less harmful to the planet. To achieve this, all stakeholders—sometimes with divergent (or even antagonistic) objectives, expectations, visions, and agendas—must actively work together to ensure the successful operationalisation of RIH principles in innovation processes within health organisations and systems.

The COVID-19 pandemic has propelled health organisations and systems into the digital age in several countries.^1-8^ During this period, digital health services have multiplied and continued to grow. By helping to limit the circulation of the virus and save lives, digital health technologies (DHTs) have proven to be crucial in many situations (Box 1).^1-4,6-8^ However, the way these technologies are designed, developed, promoted, marketed, implemented, and used in healthcare is increasingly being questioned in the literature.^1,5,9-12^ Several studies have highlighted that DHTs contributed to intensifying inequalities in access to healthcare during the COVID-19 pandemic, even in so-called public or universal health systems.^1,5,9-15^ Indeed, part of the population did not have the necessary equipment, internet connection, digital literacy, and/or linguistic skills to benefit from these services.^1,5,16^ There are also growing concerns that these inequalities, as well as the financial difficulties of health systems, will be exacerbated by the increasingly influential role of venture capital in digital health, which is characterised by high capital intensity and profit maximisation.^1,5,17-22^ Preoccupations are also increasingly expressed about the environmental impact of DHTs.^23-26^ The globalised chain of their production and supply contributes to pollution, destruction of natural ecosystems, and climate change, thus increasing the gap in health inequalities and vulnerabilities.^27,28^ DHTs’ environmental footprint is linked to, and steadily increases along the production and disposal of wearable technologies (eg, smartphones and electronic devices), the raw material needed for their production (eg, iron, aluminium, gold, mercury, and cyanide), and the energy needed to collect, manage, and store health data (eg, biometric data, artificial intelligence, and blockchain).^24,29^ Today, health data represents about 30% of total global data.^30^ Given these challenges and concerns, an emerging literature is calling for the adoption of a different innovation paradigm to ensure a responsible, equitable, inclusive, and sustainable integration of DHTs into healthcare.^1,5,22,31-34^

**Box 1. Definition of Digital Health Technologies^29,35^**

According to the WHO, digital health can be defined as “the field of knowledge and practice associated with the development and use of digital technologies to improve health (…). It includes a wider range of smart and connected devices. It also encompasses other uses of digital technologies for health such as the Internet of Things, advanced computing, big data analytics, artificial intelligence including machine learning, and robotics.”^35^

---------------

Abbreviation: WHO, World Health Organization.

###  Responsible Innovation in Health 

 Because health organisations and systems play a central role in the success or failure of the transition to more equitable and sustainable societies, Responsible Innovation in Health (RIH) has gained interest in research, policy, and practice. RIH is defined as “a collaborative endeavour wherein stakeholders are committed to clarify and meet a set of ethical, economic, social and environmental principles, values and requirements when they design, finance, produce, distribute, use, and discard sociotechnical solutions to address the needs and challenges of health systems in a sustainable way.”^9^ Rooted in the Responsible Innovation (RI) scholarship where RI means “taking care of the future through collective stewardship of science and innovation in the present,”^36^ RIH promotes the engagement of different stakeholders in scientific and technological development through participatory and inclusive approaches, in order to collectively design “ethically acceptable, socially desirable and sustainable” innovations.^36^ RIH aims to align the processes and outcomes of innovations with society’s collective needs, values, expectations, and system-level challenges.^9^ More specifically, it sheds light on the challenges of equity, justice, inclusivity, and sustainability inherent in the use of innovations in healthcare.^9^

 RIH is anchored in an “evidence-informed health research tradition.”^32,37^ It offers a critical and reflexive perspective to explore the responsibility of the increasing number of technological innovations in healthcare that are increasingly disruptive (eg, genomics, nanotechnologies, biomedical engineering) and may profoundly affect our societies.^38^ In the case of DHTs, the relevance of RIH lies with the fact that it transcends a mere deontological or ethical perspective, and rather aims to steer innovation towards equitable and economically and ecologically sustainable health systems.^32,39^

###  Goal of the Study

 Statements of principle on responsible DHTs have proliferated in recent years.^22,31-34,40-43^ However, limited empirical evidence currently exists on the operationalisation of these principles in the development, procurement, and/or utilisation of DHTs in health organisations and systems.^44,45^ There is an urgent need to address this knowledge gap. The objectives of this study are therefore to: (1) explore and understand how stakeholders perceive the inclusion of RIH principles in the development, procurement, and/or utilisation of DHTs within a health organisation; (2) identify the conditions and factors enabling and constraining the success of such an approach; and (3) stimulate a broader dialogue on the place of RIH in the upcoming “digital revolution” in health organisations and systems.

## Theoretical Framework

 We use the RIH framework developed by Silva et al,^9^ which aims to inform decisions about the degree of responsibility of health innovations and their ability to meet society’s collective needs and values. It is comprised of nine responsibility attributes grouped into five value domains: (1) population health value (health relevance; ethical, legal, and social issues [ELSIs]; health inequalities); (2) health system value (inclusiveness; responsiveness; level and intensity of care); (3) economic value (frugality); (4) organisational value (business model); and (5) environmental value (eco-responsibility).^9^ See Table 1 for definitions.

**Table 1:** The Value Domains and Attributes of Responsible Innovation in Health^9^

**Value domain**	**Attribute**	**Definitions**
Population health value	Health relevance	Refers to the respective importance of the health needs addressed by the innovation within the overall burden of disease, considering the causes of death, injury and disability and associated risk factors in the region where the intended users are located (eg, number of deaths, DALYs, YLDs, prevalence, and incidence rates).
	ELSIs	Refers to an innovation’s positive and negative impacts on the moral and sociocultural well-being of individuals and groups and to the legal and regulatory issues its use raises (eg, patient decision-aids and psychological support, laws and regulatory frameworks regarding individual rights, privacy, confidentiality, discrimination, data stewardship, stigma-reduction programs, and caregiver support).
	Health inequalities	Refers to the avoidable health status differences across individuals and groups that are associated with one’s socioeconomic status, social position, and capabilities (eg, skills, knowledge, perceived self-efficacy, and social network). Groups who suffer a greater burden of mortality and morbidity due to who they are or where they grow up, live and work are considered vulnerable.
Health system value	Inclusiveness	Refers to the degree of stakeholder engagement in the design, development, and pilot stages of an innovation. Involving at an early stage a diverse and relevant set of stakeholders through an accountable method is likely to improve an innovation.
	Responsiveness	Refers to the ability to provide dynamic solutions to existing and emerging challenges in health systems (eg, demographic shifts, epidemiologic shifts, human resources, service delivery gaps, knowledge gaps, and governance gaps).
	Level and intensity of care	Refers to the principle of subsidiarity according to which the most decentralised unit in the health system, including the patient, should be mobilised to provide the service when it is possible to do so effectively and safely. To support health system sustainability, RIH should seek to generate high-quality outcomes while reducing labour intensity (eg, community health and social care providers, reducing unnecessary interventions at the most specialised level of care of the health system).
Economic value	Frugality	Refers to the ability to deliver greater value to more people by using fewer resources such as capital, materials, energy, and labour time. Designers of frugal innovation aim to substantially reduce the costs of production and use of an innovation, focus on the core functionalities its users require and optimise its performance level considering the intended purpose and context of use.
Organisational value	Business model	Refers to the components through which an organisation creates, delivers, and captures social and economic value. A business model typically entails a tension between the redistribution of financial returns to shareholders and the provision of a high-quality innovation.
Environmental value	Eco-responsibility	Refers to a product, process or method that reduces the negative environmental impacts of an innovation, including any equipment required by its use (eg, batteries).

Abbreviations: DALYs, disability-adjusted life years; YLDs, years lived with disabilities; ELSIs, Ethical, legal, and social issues; RIH, Responsible Innovation in Health.

 The framework emphasises a collective approach that can inform decision-making about innovations, including drawing attention to the vulnerability of certain social groups and consideration of the common good that a health system represents. As Silva et al,^9^ pointed out, responsibility is not a new concept in health. However, no formal mechanisms provide the means, or at least the practical guidance, to develop and implement innovations with high societal value both in terms of equity and health system economic and environmental sustainability.^9,31-33,45^ Hence, we use this framework because it “provides a holistic, yet detailed set of dimensions through which both health and innovation policy-makers can apprehend the context of creation of RIH.”^9^

## Methods

 This study is reported in accordance with the Consolidated criteria for reporting qualitative research (COREQ) guideline.^46^ We conducted a qualitative study in a large Canadian academic health centre (hereafter called “the City hospital”), located in the province of Quebec (Box 2).^29,47,48^ The City hospital senior management team supported this study as it wanted to know how it could operationalise RIH principles in its DHT choices, and under what conditions.^29,48^

**Box 2. Description of the City Hospital**

The City hospital is one of the largest academic hospitals in the province of Quebec and the entire country (Canada). It offers specialised and sub-specialised services to adult patients. It employs over 14 000 people, including over 1000 physicians, 4000 nurses and over 2000 other healthcare professionals. It treats around 500 000 patients annually. It also houses one of the largest medical research centres in Canada, with more than 2300 staff and an academic mission to produce and disseminate knowledge and research results.

###  Recruitment

 A purposive participant sampling strategy was adopted to achieve a broad internal diversification of perspectives on the phenomenon of interest.^49^ We sought a range of participants who held responsibilities or were involved in the development, assessment, acquisition, integration, and/or utilisation of DHTs: middle and senior managers, clinicians, technology providers, health technology assessment (HTA) specialists, jurists/lawyers, patients, and researchers.

 Our research team has no professional or institutional links with the City hospital that might affect its independence (eg, in terms of the collection, analysis and publication of results). Initially, the hospital’s senior management team provided us with a list of potential participants, ie, people involved in and/or concerned by DHT-related projects and initiatives (eg, clinician and patient users, project management, technology assessment, research, purchasing, legal issues), as well as DHT companies with which the hospital collaborates and/or has service contracts. However, to ensure a broad diversity and range of viewpoints, and to limit organisational bias, an Internet search was conducted to identify technology providers and potential participants not on the initial list. Additional participants were also suggested at the time of the interviews or by people who were unable to take part in the study.^50^

 HA and PL listed potential participants and the research team then examined their profile, expertise, and perspective to ascertain whether their contribution would allow for a wide diversity of perspectives and viewpoints. This step is important to avoid a homogenous sample that might adopt a preformatted institutional discourse. With close involvement from PL, HA managed and supervised the entire study: protocol proposal, ethical review, recruitment, data collection and analysis, publication of results.

 We sent a personalised e-mail invitation to potential participants explaining the project and the reason for inviting them to an interview. Two reminders were sent in case of non-response. Of the 42 invitations sent, 29 individuals agreed to be interviewed. Table 2 summarises participants’ characteristics. Most interviewees combined various expertise, experiences and/or professional roles.

**Table 2:** Summary of the Study Participants’ Characteristics (n = 29)

**Type of Participants (Category of Respondents)**	**N**
Decision-makers/senior managers	3
Intermediate managers and clinicians-managers working on innovation and technology projects	9
Clinicians-informaticians-researchers working on innovation and technology projects	5
Procurement/contracting and jurists/lawyers specialists working on innovation and technology projects	3
Patients	4
Technology providers	5
Total	29

###  Data Collection

 Semi-structured interviews were conducted by HA between March and July 2021, in French (27) and English (2). They took place on the Zoom^TM^ videoconferencing platform, lasting between 30 and 90 minutes. Prior to the interview, participants were given a consent form summarising the study objectives and process. We obtained verbal and written consent from each participant. All interviews were audio recorded. Interview questions were guided by Silva and colleagues’ RIH framework (See Supplementary file 1).^9,29,48^ Prior to the study, the qualitative interview guide was pretested with two respondents to ensure that the questions were relevant, clear and consistent. The guide was subsequently adapted in light of emerging topics, without altering its original core structure. The interview transcripts were not returned to participants because the answers were explicit and did not necessitate any elaboration. Team members’ knowledge gained through previous involvement in multiple research projects on digital technologies and innovations in Quebec and Canadian organisations supported a contextualised triangulation of data.^5,29,51^

###  Data Analysis

 HA transcribed the interviews and then coded and analysed the data using Dedoose^TM^ software. He performed the first round of analysis and developed a preliminary coding scheme. The second round of coding and analysis was refined, discussed, and challenged by PL. The analytical process was iterative, combining a mix of deductive and inductive thematic analyses.^49,52,53^ The deductive analysis relied on the RIH framework (Table 1), whereas the inductive analysis was meant to capture emerging themes not covered by the RIH framework.^49,52,53^ However, no additional themes were identified. Saturation was reached during the analytical process where successive versions of the themes and sub-themes constituting the findings were developed, which entailed returning regularly to the whole dataset.

 The study was approved by the City hospital Research Ethics Committee (Comité d’éthique de la recherche-City hospital - 20.399).^5,29^ The letter “P” followed by a number is used in the findings to differentiate interviewees. The research team is comprised of women and men and includes researchers (MSc [Master of Science], PhD [Doctor of Philosophy]) and physician-researchers (MD [Doctor of Medicine], MPH [Master of Public Health], MBA [Master of Business Administration], MSLP [Master of Speech-Language Pathology], GCHlthMgt [Graduate Certificate in Healthcare Management]) with an extensive experience in quantitative, qualitative, and mixed methods, as well as in health innovation, digital health, HTA, program evaluation, public health, medicine, and health services and policy.^5,29^

## Results

 Our results first explore participants’ views on what responsibility in the development, procurement, and/or utilisation of DHTs entails within the City hospital and then describe how they approach the nine attributes of the RIH framework in relation to DHTs.^9^ Table 3 provides illustrative quotes for each major theme (translated by the authors from French to English when applicable).

**Table 3:** Illustrative Respondent Quotes for Each Major Theme

**Themes**	**Illustrative Quotes**
Definitions of responsible DHTs	“Is this the guarantee of a sustainable society? The ethical aspect must be part of every consideration in innovation in the whole health sector (...). The ecological dimension too. I have the impression that the more we consider the ecosystemic aspect, the more we [see that we] destroy our societies, the more we overload the health system” [P17].
Population health value	“When an industry member approaches the hospital to codevelop, validate or integrate a DHT, the first question we ask internal teams and technology developer, or providers is: To which need does the technology respond to? For patients or front-line teams*?” *[P14]. “Why we need to put 500 000 dollars in a pandemic time for something like that? Between us, it’s not a priority. So, (...) health relevance or clinical need, it’s not the first criterion considered in this project” [P8]. “We have a technology currently (...). The need is not coming from the people on the ground. Nobody came to us and said: ‘We need a new [the technology], because [the pathology] is really catastrophic (...).’ Basically, the company arrived in the field of [the pathology] (...). And then, when you arrive on the ground, and you tell the clinical teams: ‘We have a new technology for you.’ They tell us: ‘Euuh....why? We already have one. What problem do you want to solve exactly?’”[P4]. “For some patients, it may be the cost of the devices or how to purchase them too. The patient might not be able to afford the device (...). They have to know how to use them too (...). Support is important. Not leaving tools for patients to use and saying: ‘Get on with it’”[P19]. “We have a project with a single mother, with xx children, in a disadvantaged area (...). No remuneration, because we don’t pay the patients (...). We had found the patient. She said: ‘If you can help me, to make things easier, by having grocery coupons.’ [Our answer]: ‘No, we can’t.’ In this case, she has other things to do (...)” [P27].
Health system value	“Sometimes we ourselves are parachuted into projects after the project has already started. In reality, there were many stakeholders who should have been considered, but were not from the beginning. So, it’s not always systematised, and it should be” [P22]. “We’re not as responsive as we should be. It’s quite rigid. Often, we’ll put in place a technology. Then, there is no follow-up or control over time. They say: ‘It is a revolutionary technology.’ Then, they implement it. Clinicians end up not using it or it is misused (...). You must have this flexibility, for example to withdraw it. This is what creates frustration for clinicians. New ways of doing things are imposed on them, without any real follow-up or reverse listening: Experiential knowledge in the real context of use” [P26]. “We are a tertiary and quaternary centre supporting the first and second lines. We do not replace them. That [the level and intensity of care] is something we systematically consider in any digital innovation project. On the other hand: Is the health system good in this? The answer is: No. There are still issues at the health system level, particularly in terms of the intensity of care and digital innovation around that” [P14].
Economic value	“In terms of technology assessment, it is not considered. It’s obvious that we can’t spend resources, energy, and time on technologies that are not going to be viable in the long term. For me, it’s a false debate, honestly” [P13]. “It’s already very difficult to certify medical technologies. It’s even more difficult for frugal innovations. So, I don’t think frugality is going to be there soon (...). Currently, if clinicians see that there’s a problem (...). They’re going to tinker with it to make something simple to use on the ground. They won’t wait to go through the whole bureaucratic machine” [P4].
Organisational value	“Business models are one of our weaknesses. Are we also considering responsible business models when we want to move forward? We are trying to evaluate this, but we don’t have a structured framework for doing so (…). We are more and more in ‘Software-as-a-service’ business models, with licences or subscriptions per month or per year. Companies want to have money flowing in frequently” [P14]. “We are moving from sales to continuous maintenance. It’s very lucrative for technology providers. It’s very traditional business models. We had a not-for-profit organisation that came to us with [a DHT] and a model that allows them to cover their costs. So, very social enterprise. But it didn’t work out too well. The enterprise didn’t have enough resources, as a non-profit organisation, to enter a hospital” [P8].
Environmental value	“We have a big debate with the Public Procurement Authority. We had a case where two suppliers submitted their bids. Only one complied with our environmental criteria. The other supplier sued us saying that we had made the criteria very strict on purpose to destroy competition. We had to develop a whole argument and demonstrate that it was part of the City hospital’s 2019-2023 orientations where sustainable development, and environmental issues are prioritised (...)” [P23].

Abbreviation: DHTs, digital health technologies.

###  Definitions of Responsible DHTs

 When asked what responsibility in DHTs means, several interviewees referred to the definition of the “Montreal Declaration for the Responsible Development of AI [Artificial intelligence]”: “all those involved in the development of AI systems (AIS) should exercise caution by anticipating as far as possible the adverse consequences of the use of AIS and taking appropriate measures to avoid them.”^40^

 When reflecting on RIH, patients, clinicians, and managers saw technology as having to provide real added clinical value, to benefit all without discrimination, ie, ensuring equity and fairness, and to follow an open innovation strategy [P4, P8, P9, P12, P14, P19, P20, P25, P26]. Several respondents stressed that technology should also contribute to addressing the most urgent or priority needs of individuals and communities with the aim of improving the health of populations and reducing health inequalities [P8, P14, P17, P20, P25]. Some considered that technology should be subject, at the early design stage, to a collective reflection on potential future harmful or unintended consequences, and called for actions and tools to remedy this possibility [P4, P8, P14, P17, P27]. They stressed the importance of iterations throughout the technology’s life cycle to respond to the evolving realities and needs of end-users (eg, literacy, availability of up-to-date technology and equipment, high-quality internet, cognitive constraints, and cultural expectations).

 For several managers, sustainable development and the environmental and social impact of DHTs are critical [P4, P5, P8, P14, P17, P22]. Two respondents underscored that the health system is a major polluter and emitter of greenhouse gases, therefore aligned with RIH consideration of energy and resources consumption, from manufacture to end-of-life disposal (eg, extraction of rare minerals in developing countries) [P8, P17]. At the social level, an interviewee highlighted the importance of respecting human rights throughout the supply chain (eg, child labour in some countries) [P17].

 According to a manager, technology should not infringe on the integrity and freedom of individuals and communities [P25]. For patients and clinicians to understand how a diagnosis or treatment decision is being made, AI technology should be intelligible and interpretable, and its “black box” functioning should be explained and communicated easily to users. Other interviewees emphasised that a DHT should be transparent regarding the use made of the data and allow users (eg, patients, organisations) to access them at any time [P6, P8, P14, P15]. An interviewee pointed to the importance of human agency with DHTs [P25].

###  Population Health Value

####  Health Relevance

 According to several managers, health relevance is a top-priority in the City hospital’s innovation cycle [P7, P12, P13, P14]. While some DHTs are not directly aimed at meeting patients’ needs, the primary criterion for selecting a technology is precisely the needs of patients or front-line teams and added health value. For example, many AI technologies (about 72%: 62/87) implemented and/or being tested in the City hospital are intended for the diagnosis, treatment, or monitoring of complex diseases (eg, cancers, neurological diseases, eye diseases) where the overall burden of morbidity and mortality is substantial (Table 4). For several respondents, these technologies do have health relevance [P1, P3, P7, P13, P14, P21, P29].

**Table 4:** Overview of AI Technologies at the City Hospital

**Technologies and Purposes**	**Development and Experimentation**	**Implanted or in Implementation Process**	**Sustained**	**Withdrawn**
Decision-making or decision-making support: diagnosis/treatment/monitoring of patients	48	10	4	-*
Optimisation and organisation of human resources	9	4	-	-*
Administrative, material and/or logistical	3	1	-	-*
Training, learning and knowledge transfer	3	2	-	1*
Other purposes	1	1	-	-*
Digital projects, but without AI	11	13	4	-*
Total projects		115 (including 87 AI projects)		

Abbreviation: AI, Artificial intelligence. The numbers are indicative and may have changed since the time of data collection (March-July 2021). * Other technologies were reportedly discontinued or paused, but not referenced at the time of data collection.

 According to managers and clinicians, given its reputation as a leading academic hospital, the City hospital is regularly solicited to codevelop, validate and/or integrate technologies for which the health relevance is sometimes questionable [P2, P4, P5, P8, P14, P17, P26]. These requests can come from companies seeking to showcase and legitimise the value proposition or promise of their technology. It was also stressed that some pressures could be political too.

 Interviewees talked about the lack of potential health relevance of technologies, despite their suppliers’ intention to help with the management of diseases or health conditions [P4, P5, P8, P17, P26, P27]. Yet, clinical teams may simply not use them. For several clinicians and managers, this situation may arise because health relevance is mainly assessed from the patients’ perspective and less so from the clinicians’ perspective [P4, P5, P12, P17, P26]. For a respondent, technology acquisition is mainly perceived as an administrative task, which means that clinicians are not always involved in need analysis, technology design or selection [P26].

 In contrast, managers and a clinician-manager tempered this point by indicating that the health relevance of the technology may be real at the beginning, but not durable over time [P7, P13, P14, P26]. Respondents highlighted that relevance should rather be evaluated continuously through time by capitalising on the feedback and comments of frontline clinical teams [P4, P8, P13, P14, P26]. Then, one should be able to adapt or withdraw the technology if necessary.

####  Ethical, Legal, and Social Issues 

 Several interviewees talked about the difficulties they face when identifying the ELSIsassociated with DHTs [P4, P8, P12, P13, P14, P17, P25, P26]. For a respondent, the potential negative impacts on certain vulnerable and/or disadvantaged populations are often questioned by the hospital teams whenever a technology is submitted to them [P14]. This is especially important as DHTs present serious risks of violating people’s privacy and of possible bias and discrimination underlying AI technologies (eg, gender, ethnicity, and age). Nonetheless, for certain managers and a clinician-manager, instead of trying to cover all ELSIs at the beginning, which they consider impossible, mechanisms for monitoring and swiftly addressing ELSIs should be implemented in the real healthcare context [P8, P13, P14, P26]. Clinicians and managers acknowledged that the organisation does not always have the capacity to ensure that technology providers do comply with its data security and patient privacy requirements and rules [P2, P6, P8, P14, P15, P17].

####  Health Inequalities

 According to several respondents, given its publicly funded health system mandate, the City hospital should, in principle, manifest a high concern for health inequalities [P4, P8, P9, P13, P14, P17, P19, P25, P26, P27]. However, this vision is not always easy to translate into reality [P4, P8, P14, P17, P19, P25, P26, P27]. The City hospital does not have structured frameworks or mechanisms to ensure that tangible benefits from technology will arise for disadvantaged and/or vulnerable populations [P8, P13, P14, P25], and it may lack the resources and expertise to systematically examine the needs and realities of different subgroups of the population [P5, P4, P8, P13, P14, P27]. Hence, though health relevance is a key criterion, its operationalisation for disadvantaged, vulnerable or sociocultural groups appears limited. Certain participants reported that there is no legal framework to make the City hospital, as well as other health organisations in Quebec, accountable when a technology leads to, or exacerbates inequalities [P8, P17, P26, P27].

 To address such issues, the City hospital had adopted a strategy to involve patients in the assessment and choice of technologies. For several managers and patients, the organisation is a pioneer of the “patient partnership” model, ie, a collaborative approach wherein a patient who wants to be a partner in his/her care and a clinician/organisation share their complementary knowledge and perspectives on the disease and what it entails to live with it [P4, P5, P9, P14, P20, P27].^54^ However, managers and patients reported that patient involvement was sometimes seen as a mere administrative formality to justify decisions [P4, P8, P19, P20, P22, P27]. For some, patient-partners who are regularly mobilised are not necessarily representative of the populations that may be negatively impacted by technology [P8, P17, P19, P22, P27]. Two respondents highlighted that the Quebec health system operating rules, which prohibit payment to patients in non-research projects, do not facilitate the involvement of patients or citizens from vulnerable and disadvantaged groups [P14, P27]. This context favours the involvement of retired, educated, and/or middle/upper class individuals who are more likely to volunteer. In addition to putting in place financial and logistical mechanisms to encourage the involvement of representatives from vulnerable and disadvantaged groups, several patients and managers stressed the importance of building bridges between the City hospital and community organisations that can potentially help those making decisions about DHTs to better understand these groups’ realities and needs [P14, P17, P19, P20, P26, P27]. Some respondents nonetheless reported that certain stakeholders are historically reluctant to involve patients in decision-making [P5, P8, P14, P17, P19, P27]. This resistance appeared to be explained by the lack of training or preparation to collaborate on an equal basis with patients.

 According to two interviewees, the lack of consideration of inequalities is also due to the scientific literature, which has not adequately addressed these issues in DHTs [P13, P14]. They also reported that HTA is mainly based on clinical literature reviews [P13], and may not (sufficiently) address issues falling beyond the clinical domain. As a result, health inequalities may be underestimated or not considered at all. Lastly, the literature does not yet offer practical tools and methods to scientifically assess and measure inequalities.

###  Health System Value 

####  Inclusiveness

 For several interviewees, inclusiveness of all stakeholders in the whole innovation cycle is important in the City hospital [P5, P7, P14, P25]. At the outset of each project, a “stakeholder mapping” is done to determine the right stakeholders to include [P7, P14]. However, respondents acknowledged that mechanisms, processes, and/or accountability frameworks to ensure inclusiveness are not systematically used [P4, P8, P22, P26]. As a result, inclusion remains dependent on the will of the individuals leading the project, but also on the supplier with whom the City hospital co-develops it. As explained by a manager, given the rapidly changing DHT ecosystem, stakeholder inclusion is sometimes very difficult to achieve [P7].

####  Responsiveness

 Several interviewees agreed that responsiveness is not a strong point in the decisions and processes surrounding DHTs [P4, P8, P12, P14, P17, P26]. A manager underscored that the City hospital is promoting the use of the “Evidence Based Framework” for identifying the level and strength of the evidence needed to decide whether a technology satisfies requirements of effectiveness, safety, and costs [P14]. However, collecting evidence in the real-life context of care is sometimes difficult, especially in relation to frontline patients’ and clinicians’ experience of the DHT [P4, P14, P26, P27]. Respondents also reported that it is not uncommon for technologies to no longer be able to evolve to meet practical needs [P2, P4, P8, P9, P26, P27, P28].

 According to managers and clinicians, the difficulty in considering responsiveness of DHTs is also due to regulatory constraints and the rigidity of the laws that govern licensing and approval of technologies for clinical use [P2, P4, P14, P26]. Any adaptation or evolution of the technology to meet clinical needs would have to go through a complex regulatory process again, which could take several months, or even years, when additional studies are required.

####  Level and Intensity of Care

 For several interviewees, as a university hospital with a tertiary and quaternary mission, the City hospital mainly cares for patients with complex pathologies and thus mobilises highly qualified human resources and advanced technologies [P2, P4, P8, P13, P14, P16, P18]. It is therefore not surprising that many DHTs are intended for the diagnosis, management, and follow-up of complex pathologies with a high intensity of care. As managers reported, while the City hospital is willing to reduce the intensity of care when relevant, it is not easy to do [P8, P14, P26]. Firstly, the organisation is dependent upon what is available in the DHT market, which is mainly focused on selling advanced technologies in specialised care areas (eg, cancer, diabetes, and ophthalmology). Secondly, the way in which the health system is organised, governed, and financed sometimes complicates the acquisition of DHTs that allow for less intensive patient care (eg, coordination with primary care services). In this regard, a manager pointed out that funding from the Ministry of Health and Social Services (French acronym is MSSS) is mainly focused on curative specialised services to the detriment of other less intensive care modalities [P14].

###  Economic Value

####  Frugality

 Many interviewees agreed that frugality is a very poorly understood concept within the City hospital [P4, P5, P7, P8, P13, P14, P17, P22, P25]. For some, it is mainly associated with low-end, lower quality (though not necessarily poor) products, or those used in low-income countries [P4, P5, P8, P13, P17, P22]. According to two managers, frugality is widely seen by clinical and administrative teams as incompatible with the high levels of quality and reliability expected of DHTs used for clinical purposes [P8, P13]. With patients’ health and lives at stake, they cannot afford to *“play witches”* with technologies that are not viable [P13]. As a result, frugality is not a criterion considered in the evaluation of DHTs for clinical use. Other respondents also mentioned that because of its reputation as a leading university hospital—considered a showcase for innovation in Canada—the City hospital attracts mainly technology providers with a classical vision of innovation, ie, more focused on cutting-edge or disruptive technologies, and less on frugality [P4, P5, P8, P14, P17, P22, P27].

 According to clinicians and managers, even if the City hospital would like to consider frugality further, this is not always feasible [P4, P8, P12, P14, P22, P25, P26]. Health is highly regulated with a focus on effectiveness, quality, and safety. The predominance of the “evidence-based medicine” culture and the regulatory and legal requirements pose a significant obstacle when it comes to tinkering (bricolage) with certain technologies so that a wider population can benefit from them [P4, P6, P8, P14, P17, P26]. According to an interviewee, tinkering with a technology could result in the loss of the technology provider’s warranty and/or insurance coverage in the event of patient harm [P6]. However, for other interviewees, in practice, clinicians are “used to” informally modify and tinker with technologies to optimise their use (eg, extending the use of the technology to other clinical applications or patient profiles) [P4, P26]. Clinicians will not necessarily seek permission from the organisation or wait for the lengthy bureaucratic process of licensing and authorisation of technologies. Yet, participants stressed the importance for the City hospital to be able to identify small local innovations or changes, to promote them, and capitalise on their potential [P4, P8, P14, P25, P26]. To begin with, the organisation should create an environment that encourages clinicians to report tinkering and adaptations, but in a logic of continuous improvement of innovations. However, managers pointed out that the organisation cannot implement such an environment if the MSSS and/or regulatory bodies (eg, Health Canada) do not bring changes to their procedures [P4, P8, P14].

###  Organisational Value

####  Business Models 

 It was acknowledged that this element is not automatically taken into consideration when thinking about DHTs in the City hospital [P4, P7, P8, P13, P14, P22]. As a manager put it: technologies used within the City hospital are commercialised by suppliers that operate primarily within the classical economic paradigm [P8]. For example, several DHTs are commercialised by venture capital-funded start-ups whose primary objective is to find the most lucrative “exit event” possible (ie, when an investor sells his/her shares in a company to maximise profits). These companies are typically very volatile – for instance, a start-up, after being bought by an investment fund, withdrew its DHT because the needs of the City hospital were “too specific” for the technology to be cost-effective. For managers and clinicians, the business models (eg, the “Software-as-a-service -SaaS-” model where the organisation pays a monthly or annual subscription fee) of some suppliers could threaten the financial sustainability and ability of the City hospital to pay for all the technologies it needs to provide quality care to its patients [P2, P4, P8, P11, P14, P17, P23, P25]. Some respondents raised concerns that these models could lock the organisation into a dependency relationship with certain suppliers [P2, P4, P8, P17, P22]. For example, it was reported that the City hospital had to pay to access to its own data, which was exclusively “own” by a technology provider.

 As a public organisation, the City hospital is subject to laws that govern the procurement of medical technologies [P1, P2, P4, P6, P8, P11, P13, P14, P23, P24, P28]. It does not therefore have the latitude to prioritise, or favour, technology providers using alternative business models (eg, suppliers pursuing a social mission, offering freely usable and exploitable technologies, and/or applying redistributive pricing schemes) [P4, P8, P11, P14, P23].

###  Environmental Value

####  Eco-Responsibility

 There was general agreement among interviewees that eco-responsibility is not considered in the decisions surrounding DHTs within the City hospital. Incentives to do so are not yet in place, with constraints coming mainly from the MSSS [P4, P6, P8, P11, P13, P14, P22, P23]. As several interviewees reported, technology public procurement laws do not include environmental impact in their criteria [P6, P8, P11, P14, P23]. This makes it difficult for the City hospital to ask technology suppliers to comply with any environmental requirements (eg, energy consumption, recycling of electronic waste, responsible mining). Suppliers that would feel disadvantaged could complain about unfair competition to the Quebec’s Public Procurement Authority. However, for two interviewees, the City hospital has some levers that it could use to encourage suppliers to reduce the environmental impact of their DHTs [P11, P23]. For example, in the case where two suppliers offer technologies with the same clinical benefits and at the same price (which is very rare), the organisation could give a preferential margin of 10% to the supplier that aligns with internal priorities or policies (eg, compliance with environmental standards). These respondents acknowledged, however, that such a scenario is an exception rather than a rule. The City hospital’s needs are often so specific that it has no choice but to choose the only supplier available on the market. In this context, it is difficult to require the supplier to be environmentally compliant, as ultimately it is the patient’s health that comes first.

 In addition, the assessment of environmental standards compliance necessitates specialised expertise, which is not currently available within the City hospital [P8, P11, P13, P14, P23]. Comparative assessment data between the environmental impact of DHTs and current clinical practices is lacking and, given their globalised value chain, the environmental impact of DHTs is very difficult to quantify [P13, P14]. Lastly, as a manager emphasised, such assessments should be conducted by regulatory bodies and government agencies (eg, Health Canada, Quebec HTA agency) to inform decisions made by organisations like the City hospital [P8].

## Discussion

###  Summary of Key Findings 

 This study sheds light on the main perceived enablers and constrainers to the development, procurement and/or utilisation of responsible DHTs in a large teaching hospital. One of its key contributions is to highlight the significant gap between stakeholders’ awareness of RIH principles and the difficulties faced in putting them into practice. Below, we summarize four key findings identified through the RIH framework showing that the challenges of implementing RIH principles are largely organisational and systemic, with the two levels being interdependent.

 First, the health relevance of DHTs is limited by a supply-driven logic where technologies with significant marketing potential are primarily made available on the market. Then, once implanted, the health relevance of DHTs is not systematically (re)assessed to determine whether their clinical added value is fully realised or has diminished over time. The ability to anticipate, continuously (re)assess and monitor the value of DHTs (and adapt or withdraw them if necessary) necessitates expertise, financial resources, formal processes, and mechanisms within the organisation. Beyond the lack of organisational accountability frameworks (eg, legal obligation, systematic patient involvement and financial compensation), our findings showed the difficulty in addressing ethical, legal and social issues as well as health inequalities, either at the design stage or throughout the entire life cycle.

 Second, including key stakeholders’ views and requirements in the development, procurement, and/or utilisation of DHTs requires systemic change in technology regulation and assessment practices, as well as organisational culture change. Regulatory frameworks for assessing, certifying, and approving DHTs are sometimes inadequate for technologies that are designed to better meet user needs while consuming fewer resources. This is particularly true for frugal solutions, which are still widely perceived as not meeting the high quality, effectiveness, and safety standards of clinical practice. Regulatory and legal constraints also limit the scope for organisations to establish internal innovative processes enabling them to nurture and consolidate “bricolages” performed by clinicians and/or patients, and thus evolve as “learning organisations.” In addition, government funding mechanisms focus on curative, complex, and costly technologies, which require higher levels and intensity of care, making it difficult to introduce frugal DHTs designed for disease prevention, primary care, and self-management.

 Third, the way the digital technology ecosystem operates its business is a major obstacle to the development and acquisition of responsible DHTs. It hinders the emergence and growth of companies that adopt responsible business models (eg, a stakeholder-centred rather than a shareholder-centred model, reinvesting profits in the company itself, pursuing a social or environmental mission). Presently, the market is dominated by digital economy firms, mainly driven by venture capital with the aim of generating the highest and fastest possible returns on investment.^29,48^ Current business models are opaque, evolve quickly, and may threaten the financial viability of health organisations and systems.^29,48^ While biases and discriminatory issues in AI abound, these firms show no interest towards ensuring fair access to healthcare for vulnerable and disadvantaged populations.^5^

 Fourth, the environmental implications of DHTs remain largely unexplored. This is not entirely surprising in a context where they are widely seen as “solutions” in the battle against climate change.^29^ Interviewees confirmed that the expertise, tools, and frameworks for measuring and quantifying the environmental footprint of DHTs are either not promoted or unavailable. The margin of manoeuvre of organisations wishing to acquire eco-responsible DHTs is also limited by current public procurement frameworks that focus on costs.^29^

###  Contribution to the Literature

 Our study provides an original contribution to the growing literature on RIH.^22,29,31-34,45,55^ It is among the few that have examined enablers and constrainers to the integration of RIH in the DHT field. It also empirically demonstrates how Silva and colleagues’ framework can be used to account for the complexity of operationalising RIH principles in a large academic health centre.

 Our results are in line with other studies on RI and RIH. Forsberg et al,^44^ previously reported that the difficulty of operationalising RI lies, among other things, in: (1) the abstract aspect of the concept, which remains rather philosophical and lacks practical grounding in the field; (2) the lack of concrete examples of successful cases and experiences of integration and use of RI; (3) the difficulty in building a common vision on technological options among all stakeholders; (4) the adaptation challenges to facilitate communication between different actors; and (5) the lack of commonly agreed governance procedures and/or frameworks.

 Healthcare is a highly institutionalised, professionalised, and regulated sector.^45,56^ Organisations have to follow defined rules and frameworks in the execution of their mission.^57,58^ The willingness to engage in RIH can easily clash with systemic responsibilities related to compliance with professional, organisational, legal, and regulatory norms and standards.^59^ According to Rivard and Lehoux,^45^ regulatory approval processes, funding models, and the inertia that generally characterises complex health systems are obstacles to the adoption of RIH principles. Indeed, such a context affect the absorptive capacities of organisations, ie, their ability to extend and/or modify their own organisational resources and processes in support of RIH.^57,60,61^ As observed in our study, the City hospital has shown a willingness to implement and promote an organisational culture that encourages RIH. However, this dynamic is confronted with the expected role of an organisation operating in a health system (eg, legal constraint precluding remunerating patients, acquisition of eco-responsible DHTs, and selection of technology suppliers with redistributive business models).

 There may be significant epistemological differences between RIH and the medical community, where an evidence-based practice approach prevails.^62^ For Thorstensen,^62^ HTA should be more than a question of available evidence because it reflects or mirrors a certain mindset inherent in the modernisation of treatments and advanced technological innovations. This view echoes with the reluctance (and also misunderstanding) our interviewees expressed towards frugal DHTs. Such technologies are perhaps considered incompatible with the techno-optimism underlying the “stated revolution” of DHTs. To paraphrase Lehoux et al,^43^ building and consolidating the legitimacy of frugal DHTs, ie, giving them institutional value, would involve “technical work (demonstrating compliance, reliability, and effectiveness), narrative work (framing, explaining, and persuading), and political work (mobilising support, engaging public authorities, supporting them through policies).” Hence, through the lens of RIH, HTA is more likely to broaden the value definition of DHTs beyond effectiveness, safety, and costs, if it succeeds in integrating ELSIs, health inequalities, frugality, eco-responsibility, and business models considerations.^63,64^

 Responsibility is an active process that takes “place in concrete social and political spaces involving and affecting concrete actors in concrete constellations.”^65,66^ Its operationalisation necessarily implies managing trade-offs and finding societal, socio-political, legal, and normative balances.^65,67^ Our study showed the difficulty of operationalising the RIH eco-responsibility, frugality, and business model principles within the City hospital. This may be due to technological developments being driven by socio-political, regulatory, financial/economic, and ideological logics that involve a variety of stakeholders who may pursue divergent, or even antagonistic, interests.^68^ Such tensions remain relatively unexplored by RI scholars. Several authors have even criticised the “conceptual angelism” found in the RI literature that tends to ignore ideological and power issues that force actors to constantly negotiate to overcome practical and political challenges in their daily activities.^43,69,70^ Indeed, conflicting agendas, different understanding about societal challenges (eg, climate change, equity and justice, and market dynamics in health systems), and information and power asymmetries constrain stakeholder engagement when working towards RI.^9^ As observed in this study, the DHT industry is primarily market-driven and profit-maximising, which implies commercial and political influences that are in many respects beyond the control of the City hospital and of the health system. According to Lehoux et al,^71^ venture capital investments in innovation-based companies control a large part of the national capacity for technological development. In such a context, start-ups have strong incentives to reproduce rather than “innovate in innovation” as they are primarily accountable to their shareholders who expect to achieve the highest possible profit margins, in the shortest timeline.^72,73^ Thompson,^24^ also reported that addressing the environmental impact of DHTs is difficult because they result from design and marketing decisions driven by dynamics and contingencies outside the health system. Likewise, the “economy first” principle is a major obstacle to governments taking RIH seriously, especially in a context of economic crisis and globalisation (eg, international competitiveness, global markets).^74^

###  Implication for Practice and Policy

 Our study can help to better identify the practical and political issues and implications of integrating RIH principles into DHTs. The latter could improve healthcare access, quality and efficiency.^2,4,75-78^ Yet, the unprecedented rapidity of DHTs development, with potentially profound impacts at all levels of health system governance, also raises many issues.^1,5,22,34,79-84^ Meanwhile, traditional regulatory and technology assessment frameworks, as well as public policies, are falling behind in responding to this new reality. RIH provides a practice- and policy-oriented basis for ensuring that DHTs meet the objectives of the health system: to improve health in an economically and environmentally sustainable way.^32^ The Silva et al,^9^ framework provides a valuable conceptual basis for considering new dimensions in the value of DHTs. As illustrated in this study, there are numerous organisational, economic, regulatory, legal, socio-political, and even ideological challenges to overcome before effectively putting into practice all components of this framework. The RIH principles cannot be embraced in digital health without changes to the regulatory and legal frameworks. The political, media, and industry discourse on DHTs, mainly technosolutionist and focused on economic growth, should also be transformed. For example, the Quebec Minister of Economy, Innovation and Energy once described medical data as a “gold mine,” adding that “Quebec could attract pharmaceutical companies by giving them access to data from the Quebec Health Insurance Agency.” Such an orientation also appeared in the “Quebec strategy to support research and investment in innovation: 2022-2027.”^85^ With its “invent, develop, commercialise” slogan, this strategy mentions the need for “sustainable development and social innovation” and for “sustainable and inclusive innovation,” but remains mainly focused on economic growth, stimulating investment and commercialisation, creating jobs, increasing productivity, and prioritising “high-impact” technologies.^85^

 Lastly, stakeholders need clear tools and frameworks to concretely understand the value of DHTs through the lens of RIH. The tool proposed by Lehoux et al,^32^ offers a promising basis for analysing the responsibility of DHTs. With a practice-oriented objective to inform decision-making, the tool measures the degree of responsibility of DHTs according to each value domain addressed in this study, namely: (1) population health; (2) health system; (3) economic; (4) organisational; and (5) environmental.^32^ Its practical value consists in the possibility of using it in two distinct ways: (1) “as a formal evidence-informed assessment tool to measure the degree of responsibility” of DHTs; or (2) “as a design or procurement brief (or template) to explore the suitability of a given DHT solution for patient care and clinical practice, and guide its development, acquisition, implementation, or use.”^32^ This tool could be integrated into HTAs that use multi-criteria decision analysis methods to capture the global value of DHTs, thereby facilitating transparent, expansive, and flexible decision-making processes.^32,86,87^ As underscored in our study, due to the rapidly evolving ecosystem of DHTs, their degree of responsibility should be continuously (re)assessed in the real context of care.^84^ This would require processes, but also well trained and equipped personnel, allowing to better anticipate and follow the evolution of knowledge and patients’ and clinicians’ needs. Thus, being able to react and respond in a timely manner by adapting or withdrawing DHTs.

###  Strengths and Limitations

 This study has both strengths and limitations. Due to its exploratory and qualitative nature, our analysis did not aim at producing generalisable results. While findings are not intended to be transferable beyond the context of academic health centres, they may raise awareness and help share lessons with stakeholders operating in similar organisations and interested and/or concerned by RIH. Health organisations can vary considerably, especially regarding their history and culture, hence the importance of contextualising the results. The City hospital operates in a publicly funded health system, which constitutes a significant difference compared to privately funded organisations and systems. Moreover, the study was conducted in a well-established and developed health system and application of the framework would differ in health systems in low- or middle-income countries.

 The number of interviewees (n = 29) was affected by the fact that the study was conducted during the COVID-19 pandemic.^29,48^ We made great efforts to recruit a wide range of participants. However, several managers and clinicians were unable to participate due to their involvement in managing the crisis. Nevertheless, drawing on participants’ experience and expertise, we gathered a rich and detailed data corpus that has allowed us to gain an in-depth understanding of responsibility in DHTs within the City hospital. The limitation of HA acting as the main lead of the study from start to end was mitigated by ongoing critical discussions with PL.^29,48^ The adoption of a rigorous qualitative research strategy, guided by good methodological practices, and a well-defined theoretical framework has increased the reliability and credibility of our results.

## Conclusion

 Digital health innovation is in its golden age with significant progress made in recent years. Boosted during the COVID-19 crisis, DHTs are expected to play a central role in 21st century health systems. This momentum also highlights the importance and urgency of in-depth reflections and concrete actions to ensure that this digital “revolution” be carried out responsibly. DHTs will have profound repercussions on the foundations of health systems around the world and on the social contract of our societies, particularly about the values of equity, justice, and universality, as well as the future of our planet. The framework of Silva et al,^9^ offers a structured basis for initiating reflection and preparing actions on responsibility in DHTs. This study offers a first example of the conditions facilitating and constraining the integration of RIH principles into the development, procurement, and/or utilisation of DHTs. While keeping in mind the health system and setting in which the study took place, our findings are potentially relevant to the international community and will hopefully encourage the sharing of knowledge and experiences with other organisations and health systems. Lastly, given currently limited empirical studies on the subject, there is a need for more research to understand how and under what conditions RIH principles can best be integrated into innovation processes in health organisations and systems.

## Acknowledgments

 HA was supported by the In Fieri research programme (led by PL), the International Observatory on the Societal Impacts of Artificial Intelligence and Digital Technologies, and the Institute for Data Valorization (IVADO). PL’s research was funded by the Canadian Institutes of Health Research. Her research group infrastructure is supported by the Fonds de la recherche en santé du Québec (FRQ-S). SES was supported by the NIHR-funded Remote by Default 2 study (ES/V010069/1), and a BRC (Biomedical Research Centre)/ARC (Applied Research Collaboration) Fellowship focused on sustainable health care.

## Ethical issues

 The study was approved by the City hospital Research Ethics Committee (Number: Comité d’éthique de la recherche-City hospital: 20.399) – Address: 850, rue Saint-Denis Bureau, S06.230, Montréal, Québec, Canada, H2X 0A9. All methods were carried out in accordance with relevant guidelines and regulations. Informed consent was obtained from all subjects and/or their legal guardian(s).

## Conflict of interests

 Authors declare that they have no conflict of interest. KM is Associate to the President & Chief Executive Officer, Pole of Innovation, Artificial Intelligence and Projects in Health, Executive Office, CHUM. Her department supported and facilitated the conduct of the study. However, the research team had all the necessary independence throughout the study.

## Availability of data and materials

 The data that support the findings of this study are available from the corresponding author (HA) upon reasonable request. The data are not publicly available due to information that could compromise the privacy of the research participants.

## Disclaimer

 The observations and views expressed are those of the authors and not necessarily those of their organizations or funding agencies.

## Supplementary files

Supplementary file 1. Guide for Semi-structured Interviews.
