# Supplementary material for: To What Extent Can Digital Health Technologies Comply With the Principles of Responsible Innovation? Practice-and Policy-Oriented Research Insights Regarding an Organisational and Systemic Issue
Source: Int J Health Policy Manag. 2024 Oct 21;13:8061. doi: 10.34172/ijhpm.8061 (PMC11549565; doi:10.34172/ijhpm.8061)
Supplement: Supplementary file 1 — Guide for Semi-structured Interviews. [file ijhpm-13-8061-s001.pdf]

Article title: To What Extent Can Digital Health Technologies Comply With the Principles of Responsible Innovation? Practiceand Policy-Oriented Research Insights Regarding an Organisational and Systemic Issue

Journal name: International Journal of Health Policy and Management (IJHPM)

Authors' information: Hassane Alami[1,2,3,4]*, Pascale Lehoux[1,2], Sara E. Shaw[4], Marietou Niang[5], Kathy Malas[6,7], Jean-Paul Fortin[8,9]

[1]Department of Health Management, Evaluation and Policy, University of Montreal, Montreal, QC, Canada.

[2]Center for Public Health Research of the University of Montreal, Montreal, QC, Canada.

[3]Institute for Data Valorization (IVADO), Montreal, QC, Canada.

[4]Nuffield Department of Primary Care Health Sciences, University of Oxford, Oxford, UK.

[5]Université of Quebec at Rimouski, Levy, QC, Canada.

[6]Executive Office & Research Centre of The University of Montreal Hospital, Montreal, QC, Canada.

[7]Department of Entrepreneurship and Innovation, Montreal Business School (HEC), Montreal, QC, Canada.

[8]Faculty of Medicine, Laval University, Quebec, QC, Canada.

[9]VITAM Research Centre on Sustainable Health, Faculty of Medicine, Laval University, Quebec, QC, Canada.

***Correspondence to:** Hassane Alami; Email: hassane.alami@umontreal.ca

Citation: Alami H, Lehoux P, Shaw SE, Niang M, Malas K, Fortin JP. To what extent can digital health technologies comply with the principles of responsible innovation? Practice- and policy-oriented research insights regarding an organisational and systemic issue. Int J Health Policy Manag. 2024;13:8061. doi:10.34172/ijhpm.8061

Supplementary file 1. Guide for Semi-structured Interviews

Study: "Integrating the principles of responsible innovation into the selection of artificial intelligence and digital technologies in healthcare organisations: a case study in Quebec (Canada)".

INTRODUCTION

1. Could you describe your role at the City hospital[1] in a few words?

   Position and career path at the City hospital or in another organisation

2. Could you describe your specific role in the development, procurement and/or implementation of artificial intelligence (AI)[2] and digital technologies (D/AI) at the City hospital?

PERCEPTIONS OF D/AI

3. How would you describe the D/AI technologies currently used at the City hospital?

   Features, functionalities, relevance, added value, security, etc.

4. In your opinion, what are the main real or expected benefits of D/AI technologies?

   For the people concerned (patients and team), the organisation, the healthcare system, society, etc.

5. According to you, what are the risks and challenges, real or potential, of D/AI technologies?

   For the people involved (patients and team), the organisation, the healthcare system, society, etc.

DEVELOPMENT, PROCUREMENT, AND IMPLEMENTATION PROCESS

6. How would you describe the process of evaluating, selecting, developing, procuring, and implementing D/AI technologies at the City hospital? (One process at a time)

7. What were the main challenges you observed during this process?

   - *Technical challenges (e.g., lack of quality data to train algorithms, interoperability,* the City hospital *IT infrastructure)*

   - *Professional challenges (e.g., understanding and using the system, changing practices, and acquiring new expertise).*

   - *Organisational challenges (e.g., integration into clinical-administrative workflows,*

[1] Name changed for project requirements.

[2] Artificial intelligence (AI) refers to a branch of computer science that develops systems that mimic the characteristics associated with human intelligence, namely: reasoning, learning, adaptation, self-correction, sensory understanding, and interaction.

organisational preparation and readiness, clinical protocols, performance criteria, healthcare trajectories).

CONSIDERATION OF THE PRINCIPLES OF RESPONSIBLE INNOVATION IN HEALTH (RIH) IN THE DEVELOPMENT, PROCUREMENT AND/OR IMPLEMENTATION OF D/AI AT THE CITY HOSPITAL

8. I've provided you with a graphic summary of the principles of RIH (See Silva et al., 2018)[3]. But before addressing them one by one, I'd like to know what you think it means for an innovation to be called "responsible"?

   Responsibility criteria? More or less responsible innovation, and why?

9. For each attribute and domain of responsibility (see below: "IMPORTANT"), I'm going to ask you questions about: 1) whether the attribute and/or domain in question is currently taken into account for D/AI? If so, how? If not, why not? And what could be done to ensure that it is better integrated into D/AI evaluation, selection, procurement, and implementation processes?)

   IMPORTANT: address the 9 attributes of responsibility of Silva et al, (2018)[4] (health relevance; ethical, legal and social issues; inequalities; inclusiveness; responsiveness; level of care; frugality; business model; eco-responsibility) grouped into five areas, namely: 1) population health value: questions the innovation's response to the population's collective needs and its contribution to reducing health inequalities; 2) health system value : questions the extent to which the innovation provides appropriate responses to the contemporary challenges of the healthcare system; 3) economic value: questions the performance and affordability of the innovation with a view to supporting equity and sustainability; 4) organisational value: questions, among other things, the company's commercial strategies and whether it provides value for consumers and society; 5) environmental value: questions the environmental impact of the innovation.

10. According to you, how could RIH principles be considered more in the selection of these technologies by managers and clinicians? And which ones are less important or less of a priority?

11. According to you, are initiatives and actions being implemented to promote responsible D/AI innovation at the City hospital? If so, please describe them. If not, please explain.

    Technical, social, organisational, ethical, and legal solutions (based on previous answers)

12. According to you, how could the elements of the current context facilitate (or, on the contrary, constrain) the responsible development, procurement, and implementation of D/AI at the City hospital and in healthcare organisations in Quebec?

    Political context, legal framework, provincial and federal funding

[3] Silva HP, Lehoux P, Miller FA, et al. Introducing responsible innovation in health: a policy-oriented framework. Health Res Policy Syst 2018; 16:90.
[4] Silva HP, Lehoux P, Miller FA, et al. Introducing responsible innovation in health: a policy-oriented framework. Health Res Policy Syst 2018; 16:90.

Individual and organisational capacities, expertise, skills, and infrastructure

Technical support and coaching for change management

Human and financial resources, business models, etc.

13. According to you, what capacities and conditions should be developed at the City hospital to better respond to these challenges and to include more principles of responsible innovation in the criteria for selecting D/AI at the City hospital and in healthcare organisations in Quebec?
14. Could the City hospital HTA teams and procurement and supply office play a key role in this process? If so, what and how?
15. Conclusion: Are there any other points we haven't covered that you'd like to share with us?

THANK YOU
